# Camel milk *Lactococcus lactis* subsp. *cremoris*: a biocontrol agent against *Staphylococcus aureus* for fresh beef biopreservation

**DOI:** 10.3389/fnut.2025.1683200

**Published:** 2026-01-08

**Authors:** Bilal Latreche, Nabila Brahamia, Esma Bendjama, Lotfi Loucif, Ibtissem Sanah, Fairouz Djeghim, Mohammed Messaoudi, Irene Giordano, Jean-Marc Rolain, Maria D’Elia, Luca Rastrelli, Samira Becila

**Affiliations:** 1 Laboratoire de recherche en Sciences Alimentaires, Formulation, Innovation, Valorisation et Intelligence Artificielle (SAFIVIA), Institut de la Nutrition, de l’Alimentation et des Technologies Agro-Alimentaires (INATAA), Université Frères Mentouri Constantine 1, Constantine, Algeria; 2 Laboratoire de Génie Biologique, Valorisation et Innovation des Produits Agro-Alimentaires (LGBVIPA), Institut des Sciences et Techniques Appliquées (ISTA), Ain M’lila, Université Larbi Ben M’hidi, Oum El Bouaghi, Algeria; 3 Laboratory of Molecular Toxicology, Faculty of Nature and Life Sciences, University of Jijel, Jijel, Algeria; 4 Département de Technologie Alimentaire, Institut des Sciences Vétérinaires et des Sciences Agronomiques, Université Batna 1, Batna, Algeria; 5 Laboratoire de Biotechnologie des Molécules Bioactives et de la Physiopathologie Cellulaire (LBMBPC), Faculté des Sciences de la Nature et de la Vie, Université Batna 2, Batna, Algeria; 6 Équipe FNPAA, Laboratoire de Nutrition et Technologie Alimentaire (LNTA), Institut de la Nutrition, de l’Alimentation et des Technologies Agro-Alimentaires (INATAA), Université Frères Mentouri Constantine 1, Constantine, Algeria; 7 Laboratory of Research on Bioactive Products and Biomass Valorization, Department of Chemistry, Higher Normal School of Kouba (ENS), Kouba, Algiers, Algeria; 8 Department of Agricultural Science, University of Naples Federico II, Naples, Italy; 9 IHU Méditerranée Infection, MEPHI, Faculté de Médecine et de Pharmacie, Aix Marseille Université, Marseille, France; 10 Department of Pharmacy, University of Salerno, Salerno, Italy; 11 National Biodiversity Future Center (NBFC), Palermo, Italy; 12 Dipartimento di Scienze della Terra e del Mare, University of Palermo, Palermo, Italy; 13 Laboratoire de Recherche en Biotechnologie et Qualité des Aliments (BIOQUAL), Institut de la Nutrition, de l’Alimentation et des Technologies Agro-Alimentaires (INATAA), Université Frères Mentouri Constantine 1, Constantine, Algeria

**Keywords:** lactic acid bacteria, camel milk, MALDI-TOF MS, 16S rRNA gene sequencing, *Staphylococcus aureus*, biopreservation, beef

## Abstract

**Background:**

The increasing demand for natural food preservation methods has drawn attention to lactic acid bacteria (LAB) due to their antimicrobial potential. In this study, two mixed LAB cultures (MCP1 and MCP2), composed of four *Lactococcus lactis* subsp. *cremoris* strains isolated from Algerian dromedary camel milk, were evaluated for their biopreservative efficacy in fresh beef stored at 8 °C.

**Methods:**

Nineteen initial LAB isolates were screened for safety, low-temperature growth, CO₂ production, antibacterial and antioxidant activity, and inter-strain compatibility. Selected strains were identified by MALDI-TOF MS and 16S rRNA sequencing, and their acidifying power was assessed.

**Results:**

MCP2 showed a strong inhibitory effect on total aerobic mesophilic count (TAMC), with a significant 2.66 log₁₀ CFU/g reduction (−24.18%) after six days. Both cultures reduced total coliforms (TC), while MCP1 demonstrated superior antistaphylococcal activity, achieving a 5.55 log₁₀ CFU/g reduction (−50.45%) in Staphylococcus aureus after 10 days. MCP2 achieved a lesser reduction of 1.89 log₁₀ CFU/g (−17.18%).

**Conclusion:**

These findings highlight the potential of camel-derived LAB as natural protective cultures for meat biopreservation, reducing spoilage and enhancing microbial safety. Further studies should explore their effects on sensory attributes and integration with packaging technologies.

## 1 Introduction

Lactic acid bacteria (LAB) are a valuable biotechnological resource due to their recognized health-promoting properties and wide-ranging applications in the plant-based, meat, and dairy industries. Although bacterial antagonism has been known for over a century, recent years have seen renewed scientific interest in specific LAB strains for their inhibitory activity against foodborne pathogens (1–3).

In response to increasing concerns over food safety and growing consumer demand for natural, minimally processed foods free from synthetic preservatives, the development of alternative preservation strategies has become a priority. Among these, the use of microorganisms or their metabolites to suppress undesirable microbiota, prolong shelf life, and improve product safety has emerged as a promising research and industrial avenue (4, 5).

Biopreservation involves the use of microorganisms or their metabolic products to enhance food safety and extend shelf-life (3, 6). The selected microbial strains must meet strict criteria: proven safety, absence of toxic metabolites, sustained activity during storage, and no adverse impact on sensory properties. Within this context, LAB are among the most suitable candidates for replacing chemical preservatives (7–9).

Foodborne diseases remain a serious global public health challenge, with bacterial agents responsible for nearly 60% of related hospitalizations. Among these, *Staphylococcus aureus* is a well-known opportunistic pathogen capable of causing infections ranging from mild skin conditions to life-threatening diseases (10).

**Figure 1:**
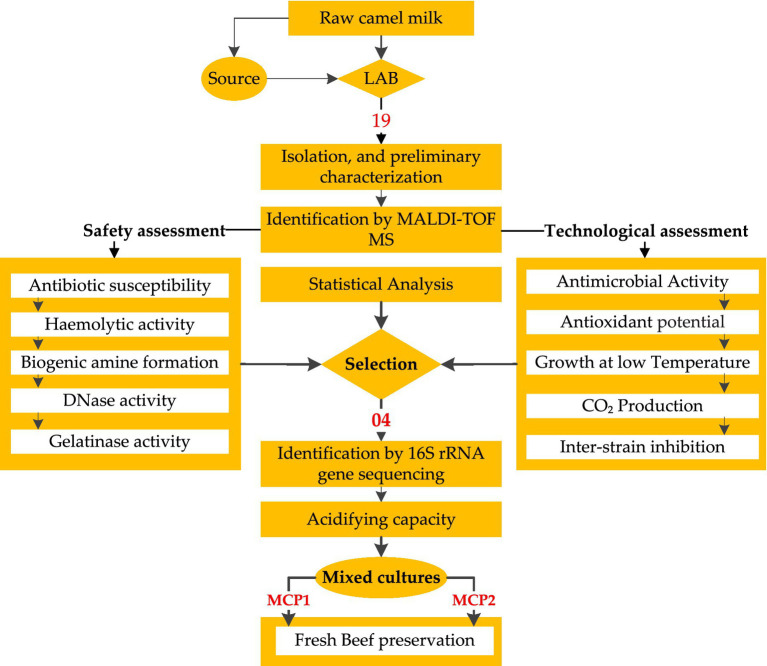
Workflow of the study design for LAB strain selection and application in fresh beef biopreservation.

Although various studies have demonstrated the antimicrobial potential of LAB and their metabolites in food preservation, particularly against key spoilage and pathogenic microorganisms these investigations have focused on distinct application scenarios. For example, (11) explored LAB consortia applied to cooked ready-to-eat meats, often in combination with non-thermal treatments, while (12) analyzed how factors like salt content and fermentable carbohydrates affect LAB-pathogen dynamics in fermented sausages (13) highlighted the broad-spectrum antimicrobial effects of LAB isolated from fermented dairy against spoilage bacteria, and (14) emphasized the role of bacteriocins and genotypic traits in controlling multidrug-resistant pathogens. However, limited research has specifically addressed LAB strains isolated from dromedary milk—an unconventional but bioactive-rich matrix, for use in meat biopreservation, particularly targeting *Staphylococcus aureus* in fresh beef. This study investigates 19 LAB strains isolated from Algerian raw camel milk for their antimicrobial activity against *Staphylococcus aureus*. Camel milk, a matrix rich in bioactive components, provides a unique ecological niche for the selection of technologically promising strains. Safety parameters such as hemolytic activity, biogenic amine production, DNase and gelatinase activity, and antibiotic susceptibility were assessed. The most suitable strains were then tested in mixed cultures for their efficacy in controlling *S. aureus* and other spoilage-associated microbial groups in fresh beef. This study aims to evaluate the practical potential of these cultures in improving the microbial safety and shelf life of perishable animal-based foods. The overall workflow of the study, from the isolation and characterization of LAB strains to the development of mixed cultures for raw beef preservation, is summarized in Figure 1.

## 2 Materials and methods

### 2.1 Raw camel milk as study material

Raw dromedary camel milk used in this study was collected from animals kept in semi-arid regions of Algeria. The milk was obtained in cooperation with local herders and camel owners, who carried out the milking using their customary methods. The collection took place immediately after routine milking sessions, and no modifications were made to normal animal handling practices. Our scientific research did not involve any direct handling of or experiments on camels and no camels were subjected to any procedures or interventions that could cause stress, pain, or harm. The milk was transported to the laboratory under refrigerated conditions and processed on the same day.

### 2.2 Isolation and preliminary characterization of LAB

The process for isolating LAB was initiated by surface plating serial decimal dilutions (from 10^−2^ to 10^−7^) on M17 (Conda, Madrid, Spain) and Man, Rogosa, and Sharpe (MRS) (Merck Millipore, Germany) agar media. The Petri dishes were subsequently incubated under aerobic conditions at 30 °C for a period of 48 to 72 h. Colonies exhibiting the typical LAB morphology were selected for preliminary characterization. Only the strains that met the criteria of Gram-positivity, catalase-negative activity, and non-motility were finally retained as presumptive LAB candidates (15).

### 2.3 Microbial strains: culture and storage conditions

After preliminary characterization, the LAB strains were subcultured at a 1% inoculum into 10 mL of MRS or M17 broth and incubated for 24 h at 30 °C. The cultures were subsequently cryopreserved at −80 °C in Eppendorf tubes containing the culture medium (MRS or M17) supplemented with 30% (v/v) glycerol. The reference strain, *Staphylococcus aureus* ATCC 25923, used as an indicator strain in antimicrobial activity assays, was cultured in Nutrient Broth (Conda, Madrid, Spain) under standard conditions (1% inoculum, 24 h at 37 °C), supplemented with 30% glycerol, and stored at −80 °C.

### 2.4 Identification of LAB strains by MALDI-TOF MS

The matrix solution was prepared by dissolving *α*-cyano-4-hydroxycinnamic acid in a mixture of 250 μL of 10% trifluoroacetic acid (TFA), 250 μL of HPLC-grade water, and 500 μL of HPLC-grade acetonitrile in a 1.5 mL microtube under a chemical fume hood. The mixture was vortexed, sonicated for 10 min, and centrifuged at 13,000 g for 5 min. The resulting supernatant was transferred to a clean microtube.

Bacterial colonies, alongside matrix-only controls and pre-characterized positive controls, were applied in thin, homogeneous layers to the MALDI-TOF MS target plate using a sterile pipette tip. Each strain was tested in triplicate. Subsequently, 1.5 μL of matrix solution was added to each spot and allowed to dry at room temperature to enable co-crystallization. After drying, the target plate was loaded into the Microflex LTII mass spectrometer (Bruker Daltonics, Bremen, Germany). Spectral data were processed following the parameters described by (16, 17). Identification confidence was expressed as a score value, with a value ≥2.0 indicating reliable species-level identification.

### 2.5 Safety evaluation of LAB

#### 2.5.1 Antibiotic susceptibility

The antibiotic susceptibility profile of the LAB strains was assessed using the standard Kirby–Bauer disk diffusion method on Mueller–Hinton (MH) agar. Briefly, sterile Petri dishes containing MH medium were prepared and solidified at room temperature. Overnight LAB cultures grown in MRS or M17 broth were adjusted to a turbidity equivalent to a 0.5 McFarland standard, corresponding to approximately 1.5 × 10^8^ CFU/mL. A 100 μL aliquot of each bacterial suspension was evenly spread over the surface of MH agar plates using sterile cotton swabs to ensure uniform lawn growth. After a brief drying period, commercial antibiotic disks were aseptically placed on the agar surface. Plates were then pre-incubated at 4 °C for 30 min to allow for initial antibiotic diffusion, followed by aerobic incubation at 30 °C for 24–48 h. Zones of inhibition were measured in millimeters (mm) and interpreted according to standard interpretive criteria: Sensitive (S) ≥ 21 mm; Intermediate (I) = 16–20 mm; Resistant (R) ≤ 15 mm, as previously described (18, 19). Each test was performed in triplicate for reproducibility. The following 13 antibiotics were tested: lincomycin (15 μg), nalidixic acid (30 μg), penicillin G (10 μg), streptomycin (30 μg), gentamicin (10 μg), ampicillin (10 μg), cephalothin (30 μg), erythromycin (15 μg), ciprofloxacin (5 μg), amoxicillin (25 μg), tetracycline (30 μg), kanamycin (30 μg), and azithromycin (15 μg).

#### 2.5.2 Haemolytic activity

The haemolytic potential of the LAB strains was evaluated to assess their safety for potential food applications. Each strain was streaked onto Columbia agar plates supplemented with 5% (v/v) defibrinated sheep blood. The plates were incubated aerobically at 30 °C for 48 h. Hemolytic activity was assessed by visual inspection of the zones surrounding bacterial colonies: *α*-hemolysis (partial hemolysis, greenish discoloration), *β*-hemolysis (complete hemolysis, clear zone), and *γ*-hemolysis (no hemolysis, unchanged medium), as previously described (20, 21). The absence of hemolytic activity is considered a key safety criterion for probiotic and food-grade LAB strains.

#### 2.5.3 Qualitative evaluation of biogenic amine formation

The capacity of LAB strains to produce biogenic amines via amino acid decarboxylation was evaluated using a qualitative screening assay. Each strain was inoculated at 1% (v/v) into five test tubes containing sterile decarboxylase broth: one control tube (no amino acid) and four test tubes supplemented at 1% (w/v) with either L-histidine, L-tyrosine, L-tryptophan, or L-phenylalanine. The pH of all broths was adjusted to 5.3 prior to incubation. Tubes were incubated at 30 °C for 48 h under aerobic conditions. Biogenic amine production was inferred from a colorimetric shift in the medium: the development of a purple coloration indicated positive decarboxylase activity and potential amine formation (22, 23). The presence of biogenic amine-producing strains may pose a food safety concern and is therefore carefully monitored.

#### 2.5.4 DNase activity

The potential of LAB strains to produce deoxyribonuclease (DNase), a virulence-associated enzyme, was assessed using DNase Test Agar (HiMedia, Mumbai, India). Each strain was streaked onto the agar surface and incubated at 30 °C for 48–72 h. After incubation, the plates were examined for clear zones surrounding the bacterial growth, which indicate DNA hydrolysis and thus positive DNase activity. The absence of a halo confirmed a negative result (24). This assay provides important insight into the safety profile of candidate strains, as DNase production is generally undesirable in probiotic or food-grade bacteria.

#### 2.5.5 Gelatinase activity

Gelatinase production, another safety-relevant trait, was evaluated by spotting 10 μL of overnight LAB cultures onto a solid gelatinase test medium composed of: 5 g/L peptone, 3 g/L yeast extract, 30 g/L gelatin, and 15 g/L agar, adjusted to pH 7.0. The inoculated plates were incubated at 30 °C for 48 h, after which they were overlaid with a saturated ammonium sulfate solution (550 g/L). The development of a clear halo around the colonies indicated positive gelatinase activity, reflecting the strain’s ability to hydrolyze gelatin (25). Absence of halo formation was interpreted.

### 2.6 Antimicrobial activity

The antimicrobial activity of the LAB strains was evaluated against *Staphylococcus aureus* ATCC 25923 using the agar well diffusion assay (WDA). LAB cultures were initially grown in MRS or M17 broth and incubated at 30 °C for 24 h. Subsequently, 2 mL of each culture was transferred into fresh medium and incubated under the same conditions for an additional 18 h to ensure active metabolite production.

After incubation, the cultures were centrifuged at 5000 rpm for 10 min at 4 °C, and the supernatants were carefully collected (23, 26). These cell-free supernatants were used as crude antimicrobial preparations.

For the WDA, *S. aureus* was adjusted to a turbidity equivalent to 0.5 McFarland standard and uniformly spread onto the surface of Mueller-Hinton (MH) agar plates using sterile swabs. After drying for 30–45 min at room temperature, 6 mm diameter wells were aseptically bored into the agar, and 100 μL of each LAB supernatant was added per well. Plates were pre-incubated at 4 °C for 4 h to facilitate radial diffusion of bioactive compounds, then incubated at 30 °C for 24–48 h (27, 28).

Antimicrobial activity was assessed by measuring the diameter of the inhibition zone surrounding each well. Zones exceeding 2 mm in diameter were considered indicative of positive inhibitory activity (29). All assays were conducted in triplicate to ensure reproducibility.

### 2.7 Antioxidant potential by ABTS method

The antioxidant potential of the LAB strains was evaluated using the ABTS radical scavenging assay, as previously described by (30), with slight modifications. LAB strains were cultured in MRS or M17 broth and incubated at 30 °C for 18 h. The bacterial cells were harvested by centrifugation at 4000 rpm for 5 min at 4 °C, washed twice with sterile phosphate-buffered saline (PBS), and resuspended in PBS to reach an optical density of 1.0 at 600 nm (OD₆₀₀).

The ABTS radical cation (ABTS^+^) was generated by mixing 7 mM ABTS with 2.45 mM potassium persulfate (K₂S₂O₈), followed by incubation in the dark at room temperature for 16 h. Before use, the ABTS^+^ solution was diluted with distilled water to an absorbance of 0.70 ± 0.01 at 734 nm.

For the assay, 300 μL of the LAB cell suspension was mixed with 600 μL of the ABTS^+^ solution in amber tubes. The mixture was vortexed for 30 s and allowed to react at room temperature for 10 min. The absorbance was then measured at 734 nm using a UV–Vis spectrophotometer.

The radical scavenging activity (RSA) was calculated using the following Equation 1:

RSA(%)=[1−(A_sample/A_control)]×100 (1)

Where:

A_sample = absorbance of the sample.

A_control = absorbance of the ABTS^+^ solution without bacterial cells.

All measurements were performed in triplicate.

### 2.8 Low-temperature growth and CO₂ production

The psychrotrophic growth capacity of LAB strains was evaluated by incubating the cultures in MRS or M17 broth at 8 °C for 12 days. Bacterial growth was assessed visually by monitoring turbidity in the culture tubes and comparing it to a non-inoculated control incubated under the same conditions.

To assess the ability of LAB to produce carbon dioxide (CO₂) from glucose, a modified MRS broth, lacking ammonium citrate and meat extract and supplemented with glucose, was used. A Durham tube was placed inverted within the test tubes to trap any gas produced. The inoculated tubes were incubated at 30 °C for 24 to 48 h. The presence of gas bubbles within the Durham tube was interpreted as a positive indicator of CO₂ production through heterofermentative metabolism (31, 32).

### 2.9 Inhibitory interactions between LAB strains

The inhibitory interactions among LAB strains were evaluated using a modified Kirby-Bauer disk diffusion method. Sterile paper disks were impregnated with 10 μL of each active LAB culture and placed on MRS or M17 agar plates previously inoculated with the indicator LAB strain, standardized to a 0.5 McFarland turbidity. The plates were incubated at 30 °C for 24 to 48 h. Antagonistic activity was assessed by the presence of a clear inhibition halo surrounding the disks, indicating inter-strain inhibitory effects.

### 2.10 Identification of selected LAB by 16S rRNA gene sequencing and phylogenetic analysis

Based on preliminary screening, selected LAB strains were subjected to molecular identification through 16S rRNA gene sequencing. Genomic DNA was extracted using the DNAeasy® UltraClean® Microbial Kit (Qiagen, Milan, Italy) following the manufacturer’s instructions. The 16S rRNA gene was amplified using the Phusion™ High–Fidelity DNA Polymerase Kit (ThermoScientific, Monza, Italy) with the universal primer pair: forward (FD) 5’-AGAGTTTGATCCTGGCTCAG-3′ and reverse (RD) 5’-AAGGAGGTGATCCAGCC-3′.

PCR amplification was carried out in a Mastercycler® Nexus Gradient thermal cycler (Eppendorf, Milan, Italy) under the following conditions: initial denaturation at 95 °C for 3 min; 35 cycles of denaturation at 94 °C for 1 min, annealing at 54 °C for 45 s, and elongation at 72 °C for 2 min; followed by a final extension at 72 °C for 7 min. Amplicons were resolved by agarose gel electrophoresis (1.5% w/v, 80 V, 30 min) to confirm amplification specificity. PCR products were purified using the QIAquick® PCR Purification Kit (Qiagen, Milan, Italy) and sequenced by Eurofins Genomics (Milan, Italy).

The resulting sequences were analyzed using the NCBI BLAST nucleotide database and compared with reference sequences for taxonomic identification. Phylogenetic relationships and evolutionary distances were computed using MEGA version 11.0 software (33, 34).

### 2.11 Acidifying capacity of selected LAB strains

The acidification potential of selected LAB strains was evaluated in UHT skim milk. Overnight cultures of each isolate were grown in MRS or M17 broth at 30 °C. Subsequently, 1% (v/v) of each active culture was inoculated into sterile flasks containing UHT skim milk and incubated at 30 °C. The pH of the milk was measured at defined time intervals (0, 2, 4, 6, 8, 24, and 48 h) using a calibrated pH meter (Mettler Toledo, SevenCompact S220, Canada). All measurements were conducted in triplicate, and results were expressed as mean pH values over time (35).

### 2.12 Raw meat preservation assay

Two distinct LAB-based microbial consortia (MCP1 and MCP2), each composed of four high-performing strains selected from preliminary screenings, were formulated to assess their biopreservative efficacy on fresh beef. The experimental protocol was adapted from (36), with minor methodological modifications.

Fresh beef filets were procured from a local butcher shop, rinsed with sterile distilled water, and air-dried under aseptic conditions. Using sterilized tools under a laminar flow hood, the filets were cut into uniform slices (~70 g; 6 cm × 3 cm × 1 cm).

LAB cultures were first activated in M17 broth at 30 °C for 24 h. After this initial incubation, 2 mL of each culture was transferred to fresh M17 broth and incubated again for 18 h. The cultures were then diluted in tryptone-salt broth to achieve a final density of 10^8^ CFU/mL. To eliminate residual media components, the suspensions were centrifuged at 8,000 × g for 10 min, and the bacterial pellets were resuspended in an equal volume of sterile tryptone-salt solution.

*Staphylococcus aureus* (ATCC 25923) was cultured in Mueller-Hinton broth at 37 °C for 24 h, and diluted in tryptone-salt to reach 10^6^ CFU/mL.

Four experimental batches were prepared:

Batch 1 (Negative control): No microbial inoculation.

Batch 2 (Pathogen control): Slices were sprayed with 1% (v/w) of *S. aureus* suspension (~10^4^ CFU/g).

Batch 3: Slices were first inoculated with 1% (v/w) of *S. aureus* suspension, followed by 1 h incubation at 4 °C to allow bacterial adhesion. Subsequently, 1% (v/w) of MCP1 suspension was applied (~10^6^ CFU/g).

Batch 4: As in batch 3, but MCP2 was used instead of MCP1.

All meat samples were incubated in sterile Petri dishes at 8 °C for 10 days. Microbiological analyses were performed on days 0, 3, 6, and 10.

To guarantee microbiological integrity, prevent cross-contamination, and ensure the validity of biological replicates, the initial meat sample was subdivided into distinct aliquots of identical mass. Each aliquot was specifically allocated for the analyses corresponding to the different time points defined by the protocol. This procedure ensured the uniqueness of each analyzed portion while maintaining its single origin. The experimental rigor relies on a combination of technical repetitions and biological replicates, the latter being precisely defined as portions of meat prepared and analyzed completely independently on distinct days of the study.

### 2.13 Microbiological analysis

The microbiological quality of meat samples was assessed by enumerating specific microbial groups using selective culture media, in accordance with internationally recognized protocols. Total aerobic mesophilic count (TAMC) was performed using Plate Count Agar (PCA). Total coliforms (TC) were determined on Violet Red Bile Lactose (VRBL) agar. Lactic acid bacteria (LAB) were enumerated using M17 agar, while *Staphylococcus aureus* was quantified on Chapman agar. The incubation conditions and media specifications for each microbial group are provided in Table 1.

**Table 1:** Culture media and growth conditions used for the enumeration of bacterial groups.

Bacterial group	Culture medium	Growth conditions
Total aerobic mesophilic count	PCA (Scharlau, Barcelona, Spain)	72 h at 30 °C
Total coliforms	VRBL (TM Media, Rajasthan, India)	24 h at 37 °C
*Staphylococcus aureus*	Chapman (Condalab, Madrid, Spain)	48 h at 37 °C
Lactic acid bacteria	M17 (Condalab, Madrid, Spain)	72 h at 30 °C

Colony-forming units (CFU) per gram of sample were calculated based on plates yielding between 30 and 300 colonies. The enumeration followed the standard equation:

N=∑C/V(n1+0.1n2).d₁

Where:

*N* = Number of microorganisms per gram of sample.

∑C = Total number of colonies counted on plates from two successive dilutions.

V = Volume of inoculum plated (in mL).

n₁ = Number of plates from the first dilution.

n₂ = Number of plates from the second dilution.

d₁ = Dilution factor of the first selected dilution.

All analyses were conducted in triplicate for each time point and treatment condition.

### 2.14 Statistical analysis

Statistical analyses were performed using methods appropriate to the study objectives. Descriptive statistics were initially computed to summarize key variables. The assumption of normality was verified using the Shapiro–Wilk test. One-way analysis of variance (ANOVA) was applied to evaluate differences among mean values, followed by Tukey’s HSD post-hoc test for multiple comparisons. For categorical variables, the Chi-squared test (χ^2^) was used. Hierarchical cluster analysis (HCA), based on Ward’s linkage method, was conducted to classify the 19 LAB strains based on phenotypic and functional traits. All statistical analyses were performed using JMP® Trial Version 17.0 (SAS Institute Inc., Cary, NC, USA). Data are presented as mean ± standard deviation (SD), and *p* values < 0.05 were considered statistically significant.

## 3 Results

### 3.1 Identification of LAB strains by MALDI-TOF MS

A total of 19 Gram-positive, catalase-negative presumptive lactic acid bacteria (LAB) were isolated from Algerian dromedary camel milk samples cultured on MRS and M17 agar. These isolates were subjected to identification using matrix-assisted laser desorption/ionization time-of-flight mass spectrometry (MALDI-TOF MS), which enabled rapid species-level classification based on proteomic fingerprints. The MALDI-TOF MS analysis yielded identification scores ranging from 2.23 to 2.46, indicating a high level of confidence in species assignment. According to Bruker’s interpretation standards, 78.95% of isolates showed a Log(score) ≥ 2.3, corresponding to secure species-level identification, while 21.05% of strains fell within the 2.0–2.3 range, suggesting probable species-level identification. Three LAB species were identified among the isolates: *Lactococcus lactis*, *Lactobacillus lactis*, and *Enterococcus italicus*. *Lactococcus lactis* emerged as the dominant species, representing 89.47% of all isolates.

These results confirm the predominance of *L. lactis* in the camel milk microbiota and highlight the utility of MALDI-TOF MS for high-throughput and accurate taxonomic screening in food microbiology.

### 3.2 Safety evaluation of LAB strains

#### 3.2.1 Antibiotic susceptibility

The antibiotic resistance profile of the 19 LAB strains was assessed via the disk diffusion method. The susceptibility patterns varied depending on the antibiotic tested (Supplementary Table 1). Notably, between 63 and 84% of strains exhibited full or intermediate susceptibility to lincomycin, penicillin, ampicillin, tetracycline, and azithromycin. In contrast, gentamicin resistance was observed in 79% of isolates, suggesting potential intrinsic resistance mechanisms common among dairy LAB. Additional antibiotics, nalidixic acid, streptomycin, cephalothin, erythromycin, ciprofloxacin, amoxicillin, and kanamycin, elicited sensitive or moderately sensitive responses in 42–58% of strains.

#### 3.2.2 Hemolytic activity

Hemolytic behavior, a critical safety trait for probiotic or food-associated LAB, was negative in most isolates. Only two strains (CMLAB18 and CMLAB19) demonstrated *α*-hemolytic activity, while the remaining 89.47% were non-hemolytic, confirming their suitability for safe application in food systems (Table 2).

**Table 2:** Identification of lactic acid bacteria (LAB) isolates from Algerian dromedary camel milk by MALDI-TOF MS, and safety evaluation based on enzymatic activity and biogenic amine production.

Strains	MALDI-TOF MS identification		Safety evaluation							Growth at low temperature	CO₂
			DNase activity	Haemolytic activity	Gelatinase activity	Biogenic amine formation					
	Result	Log (score)				*Histidine*	*Tyrosine*	*Tryptophan*	*Phenylalanine*		
CMLAB1	*Lactococcus lactis*	2.37	−	−	−	−	−	−	−	+	−
CMLAB2	*Enterococcus italicus*	2.32	−	−	−	−	−	−	−	+	−
CMLAB3	*Lactococcus lactis*	2.44	−	−	−	−	−	−	−	−	−
CMLAB4	*Lactococcus lactis*	2.46	−	−	−	−	−	−	−	+	−
CMLAB5	*Lactococcus lactis*	2.32	−	−	−	−	−	−	−	±	−
CMLAB6	*Lactococcus lactis*	2.35	−	−	−	−	−	−	−	±	−
CMLAB7	*Lactococcus lactis*	2.44	−	−	+	−	−	−	−	±	−
CMLAB8	*Lactococcus lactis*	2.39	−	−	−	−	−	−	−	+	−
CMLAB9	*Lactococcus lactis*	2.29	−	−	−	−	−	−	−	+	−
CMLAB10	*Lactococcus lactis*	2.32	−	−	−	−	−	−	−	+	−
CMLAB11	*Lactococcus lactis*	2.42	−	−	−	−	−	−	−	+	−
CMLAB12	*Lactococcus lactis*	2.38	−	−	−	−	−	−	−	+	−
CMLAB13	*Lactococcus lactis*	2.37	−	−	−	−	−	−	−	±	−
CMLAB14	*Lactococcus lactis*	2.29	−	−	−	−	−	−	−	−	−
CMLAB15	*Lactococcus lactis*	2.41	−	−	−	−	−	−	−	+	−
CMLAB16	*Lactococcus lactis*	2.34	−	−	−	−	−	−	−	±	−
CMLAB17	*Lactococcus lactis*	2.23	−	−	+	−	+	−	−	±	−
CMLAB18	*Lactobacillus lactis*	2.32	−	+	−	−	−	−	−	+	−
CMLAB19	*Lactococcus lactis*	2.27	−	+	+	−	+	−	−	+	−

Symbols: (−) negative test, (+) positive test, (±) little growth.

Additional traits assessed include growth at low temperature and CO₂ production. Identification log(scores) ≥ 2.3 indicate secure species-level assignment.

#### 3.2.3 Biogenic amine formation

All strains tested negative for histidine, tryptophan, and phenylalanine decarboxylase activity, as evidenced by the absence of color changes or typical degradation halos. However, two strains (CMLAB17 and CMLAB19) showed positive tyrosine decarboxylase activity, with characteristic purple pigmentation in the medium, indicating a limited capacity for biogenic amine production (Table 2).

#### 3.2.4 DNase and gelatinase activities

None of the isolates produced deoxyribonuclease (DNase) under the tested conditions (48–72 h, 30 °C), supporting their non-pathogenic nature. In terms of proteolytic activity, gelatinase production was limited to three isolates: CMLAB7, CMLAB17, and CMLAB19, as shown by clear zones in gelatin-containing media (Table 2).

#### 3.2.5 Statistical evaluation

Statistical analysis did not reveal any significant differences (*p* > 0.05) in the frequency of safety-related traits across the tested strains. This homogeneity in safety parameters supports the potential use of most isolates as candidate starter or protective cultures.

### 3.3 Antimicrobial activity

**Figure 2:**
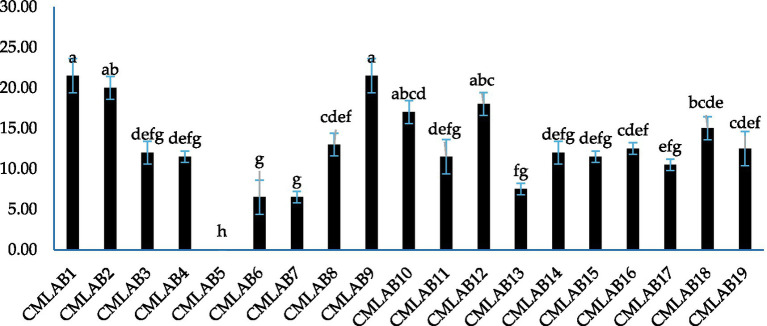
Antimicrobial activity of LAB strains against *Staphylococcus aureus* ATCC 25923 assessed using the agar well diffusion assay. Bars represent mean inhibition zone diameters ± standard deviation. Columns not sharing a common superscript letter differ significantly (*p* < 0.05).

The agar well diffusion assay (WDA) used to evaluate the antimicrobial activity of LAB strains revealed highly heterogeneous inhibitory activity. This activity was statistically significant (*p* < 0.0001) against the pathogen *Staphylococcus aureus* ATCC 25923. Overall, 94.74% of the tested LAB strains exhibited antimicrobial activity, with inhibition zone diameters ranging from 6.5 ± 2.12 mm to 21.5 ± 2.12 mm. Only strain CMLAB5 showed no inhibition (Figure 2). Strains CMLAB1 and CMLAB9 demonstrated the most significant inhibitory effects.

### 3.4 Antioxidant potential

**Figure 3:**
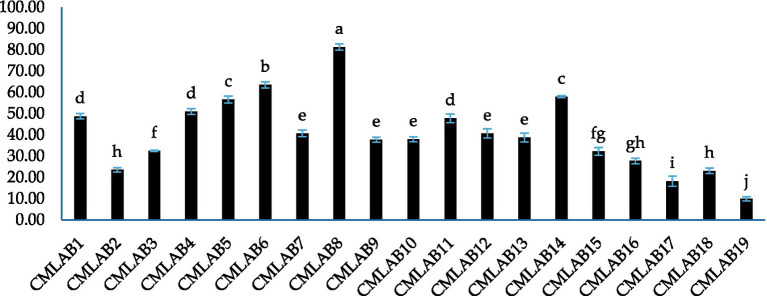
ABTS radical scavenging activity (%) of LAB strains. Values represent mean ± standard deviation (SD). Bars not sharing a common superscript letter differ significantly (*p* < 0.05).

The antioxidant activity of the bacterial strains was quantified by evaluating their capacity to reduce the ABTS radical cation. The results of this analysis demonstrated statistically significant heterogeneity (*p* < 0.0001). All isolates exhibited inhibition of ABTS radicals, with activity ranging from 9.9 ± 1.0% to 81.3 ± 1.5% (Figure 3).

### 3.5 Low-temperature growth, CO₂ production, and inter-strain inhibition

To select the most promising LAB candidates for subsequent application in meat biopreservation, qualitative evaluations were conducted focusing on growth behavior at low temperatures, CO₂ production, and antagonism between strains. Results revealed that 57.89% of the isolates were capable of growing at 8 °C in M17 broth over a 12-day incubation period, a key trait for efficacy under refrigerated storage conditions commonly used in meat preservation (Table 2).

Interestingly, all strains lacked carbon dioxide-generating metabolic activity after 24 to 48 h of incubation at 30 °C, as evidenced by the absence of gas bubbles or culture medium disturbance. This result suggests that all isolates are homofermentative and therefore unlikely to cause unwanted package swelling during storage. Such a feature is particularly beneficial in vacuum-packed or modified atmosphere packaging systems, where CO₂ accumulation can negatively impact product stability and consumer acceptance. The inter-strain antagonism assay revealed notable heterogeneity among the strains in their ability to inhibit each other (Table 3). This variability was considered during the selection process, as excessive antagonism could impair the synergistic functionality of LAB consortia. Consequently, strains with both low-temperature growth capability and mutual compatibility were prioritized for further biopreservation studies.

**Table 3:** Inter-strain antagonistic activity of lactic acid bacteria (LAB) isolates assessed by spot-on-lawn assay.

Strains	CMLAB1	CMLAB2	CMLAB3	CMLAB4	CMLAB5	CMLAB6	CMLAB7	CMLAB8	CMLAB9	CMLAB10	CMLAB11	CMLAB12	CMLAB13	CMLAB14	CMLAB15	CMLAB16	CMLAB17	CMLAB18	CMLAB19
CMLAB1		−	−	±	+	−	−	−	−	−	−	−	+	+	−	+	+	+	+
CMLAB2	−		−	−	+	−	+	+	+	+	−	±	−	−	+	−	−	+	−
CMLAB3	−	−		−	+	+	−	±	−	+	−	+	−	−	+	−	−	−	−
CMLAB4	±	−	−		+	+	−	−	−	−	−	+	+	−	−	−	−	−	−
CMLAB5	+	+	+	+		−	−	+	−	−	−	+	−	−	−	−	−	−	−
CMLAB6	−	−	+	+	−		−	±	±	−	±	−	−	+	−	±	+	−	±
CMLAB7	−	+	−	−	−	−		−	−	−	−	−	−	−	+	−	−	+	+
CMLAB8	−	+	±	−	+	±	−		−	−	−	+	−	+	−	−	±	−	−
CMLAB9	−	+	−	−	−	±	−	−		−	−	−	±	−	−	−	+	−	−
CMLAB10	−	+	+	−	−	−	−	−	−		−	−	−	+	−	−	±	+	−
CMLAB11	−	−	−	−	−	±	−	−	−	−		+	−	−	+	+	−	−	−
CMLAB12	−	±	+	+	+	−	−	+	−	−	+		−	−	+	−	−	−	−
CMLAB13	+	−	−	+	−	−	−	−	±	−	−	−		−	−	−	−	−	−
CMLAB14	+	−	−	−	−	+	−	+	−	+	−	−	−		−	−	−	−	−
CMLAB15	−	+	+	−	−	−	+	−	−	−	+	+	−	−		−	−	−	−
CMLAB16	+	−	−	−	−	±	−	−	−	−	+	−	−	−	−		−	−	−
CMLAB17	+	−	−	−	−	+	−	±	+	±	−	−	−	−	−	−		−	−
CMLAB18	+	+	−	−	−	−	+	−	−	+	−	−	−	−	−	−	−		−
CMLAB19	+	−	−	−	−	±	+	−	−	−	−	−	−	−	−	−	−	−	

Symbols indicate antagonistic effects of each strain (row) against others (columns): (−) no inhibition; (±) moderate inhibition; (+) clear inhibition. Black-filled cell: test not performed; the same strain.

### 3.6 Selection and screening of LAB strains

**Figure 4:**
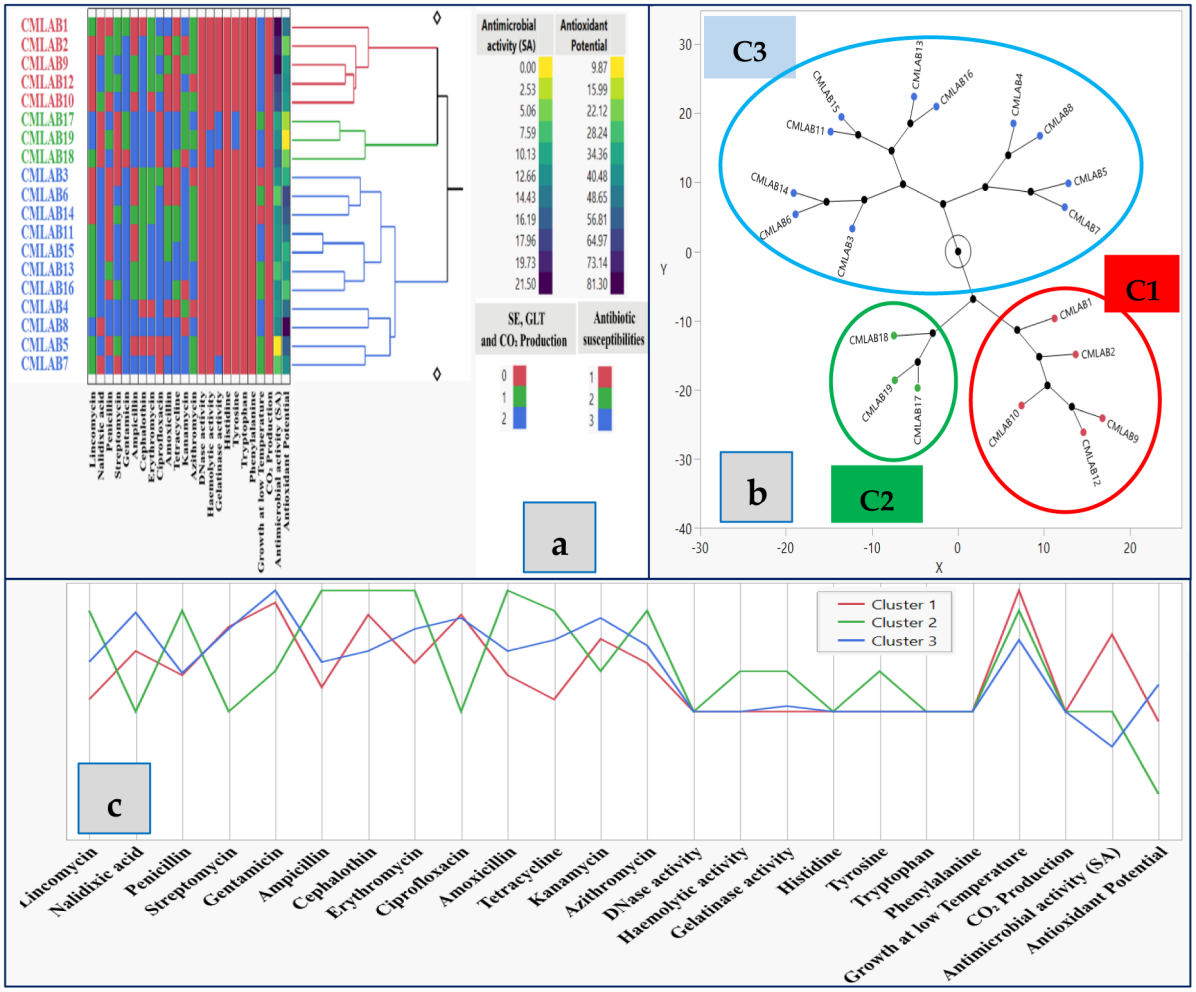
Hierarchical clustering **(a)**, constellation plots **(b)**, and standardized trait profiles **(c)** of 19 LAB strains isolated from raw dromedary camel milk using the Ward method. Cluster 1 (C1) includes strains CMLAB1, CMLAB2, CMLAB9, CMLAB10, and CMLAB12; Cluster 2 (C2) includes CMLAB17, CMLAB18, and CMLAB19; Cluster 3 (C3) includes CMLAB3, CMLAB4, CMLAB5, CMLAB6, CMLAB7, CMLAB8, CMLAB11, CMLAB13, CMLAB14, CMLAB15, and CMLAB16. Level increase is represented in blue, while level decrease is shown in yellow. Antibiotic susceptibility is coded as follows: 1 = sensitive, 2 = intermediate, 3 = resistant. SE, safety evaluation; GLT, growth at low temperature; CO₂, carbon dioxide production. CO₂ and safety traits were numerically coded as 0 (negative), 1 (slight growth or borderline activity), or 2 (positive test).

Hierarchical Cluster Analysis (HCA) of the phenotypic and functional traits of the 19 LAB strains identified three major clusters (Figure 4). Cluster 1 included strains CMLAB1, CMLAB2, CMLAB9, CMLAB10, and CMLAB12, which shared desirable traits for biopreservation applications, including high antimicrobial and antioxidant activity, safety profile, and growth at low temperatures. However, strain CMLAB2 was excluded from further development due to its relatively low antioxidant capacity. Based on this screening, two mixed cultures were formulated for potential food applications: MCP1 (CMLAB1 and CMLAB10) and MCP2 (CMLAB9 and CMLAB12), representing robust candidates for further *in situ* testing in meat biopreservation.

### 3.7 16S rRNA gene sequencing for the identification of selected LAB

**Figure 5:**
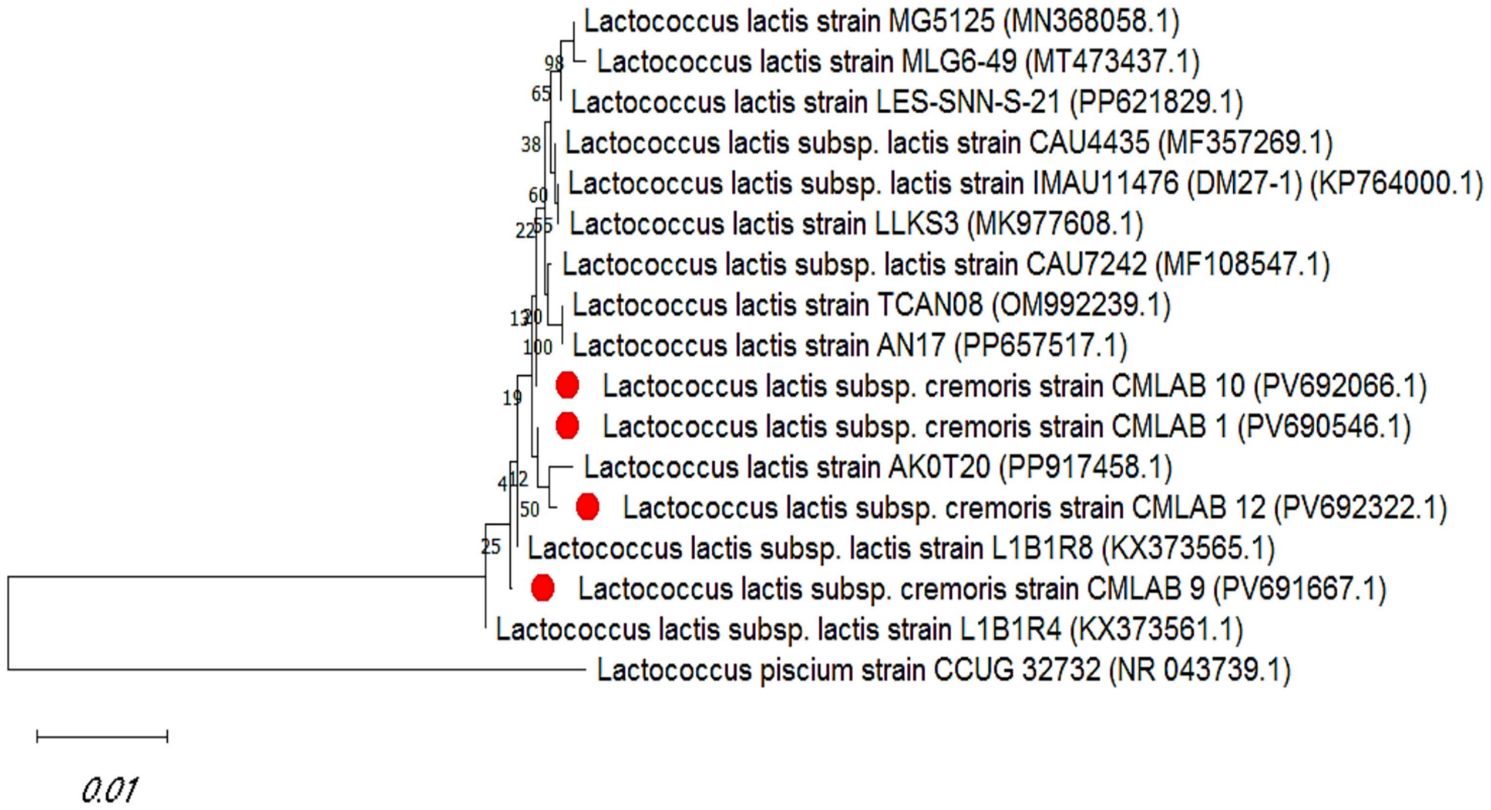
Phylogenetic tree based on 16S rRNA gene sequences illustrating the evolutionary relationships of the selected LAB strains (CMLAB 1, 9, 10, and 12; highlighted in red) with reference *Lactococcus* species. The tree was constructed using the neighbor-joining method, and *Lactococcus piscium* CCUG 32732 was used as an outgroup to root the tree. Bootstrap values (>50%) based on 1,000 replicates are shown at the branch nodes. Scale bar indicates 0.01 nucleotide substitutions per site.

The identification of selected lactic acid bacteria (LAB) strains was achieved through 16S rRNA gene sequencing. The obtained sequences were compared against the NCBI BLAST nucleotide database, enabling taxonomic assignment based on sequence homology. The four strains, namely CMLAB1 (PV690546.1), CMLAB9 (PV691667.1), CMLAB10 (PV692066.1), and CMLAB12 (PV692322.1), were all identified as belonging to the species *Lactococcus lactis* subsp. *cremoris*, exhibiting high sequence similarity with the closest reference strains available in the BLASTn database. For phylogenetic tree construction, *Lactococcus piscium* (CCUG 32732.1) was employed as an outgroup. This species, although phylogenetically related to *L. lactis* subsp. *cremoris*, provided sufficient genetic divergence to ensure accurate tree rooting and robust evolutionary inference (Figure 5).

### 3.8 Acidifying capacity of selected LAB strains

**Figure 6:**
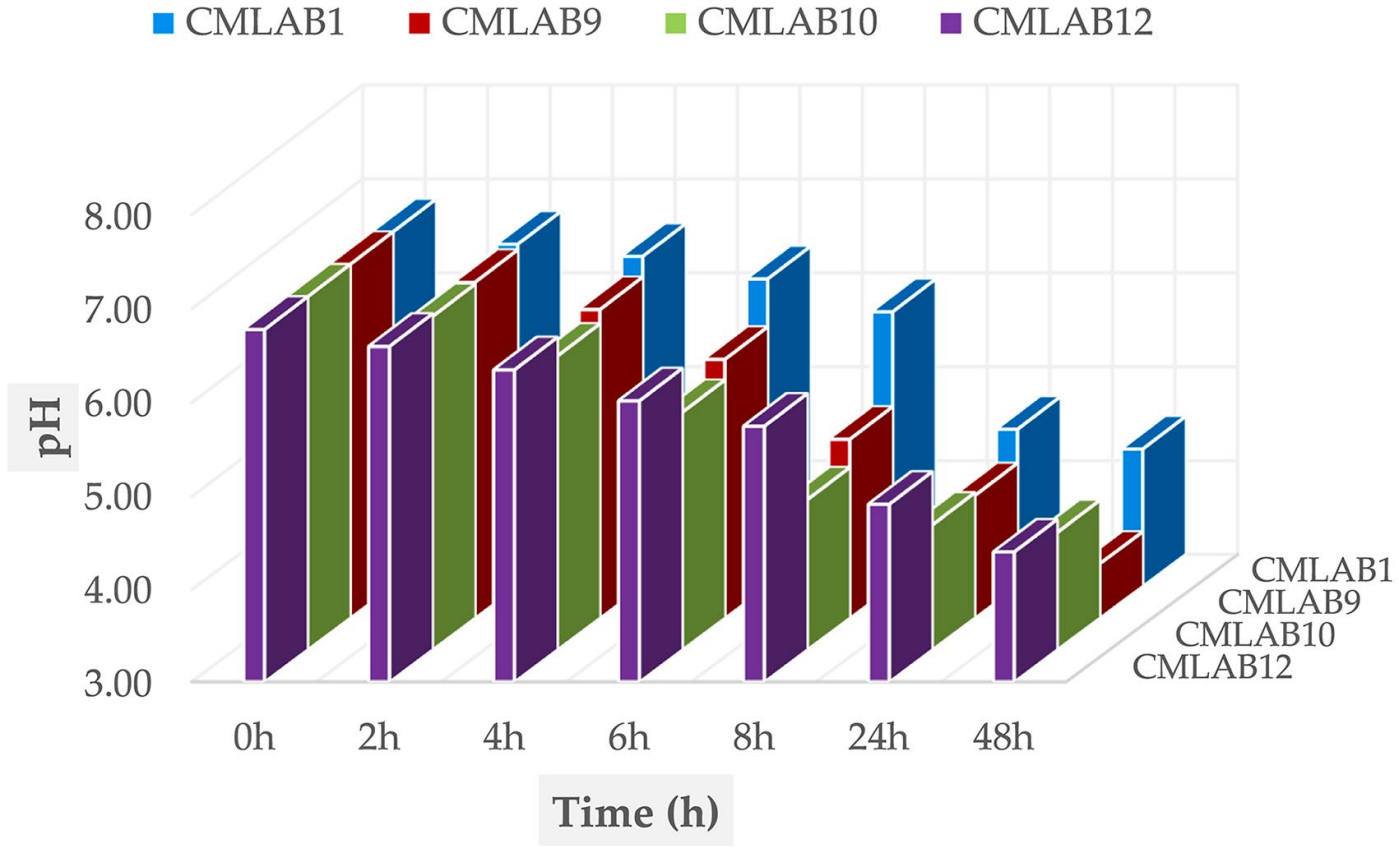
Acidifying capacity of selected *Lactococcus lactis* subsp. *cremoris* strains.

Figure 6 displays the acidification kinetics of the selected *Lactococcus lactis* subsp. *cremoris* strains over a 48-h incubation period at 30 °C. A statistically significant difference in acidification capacity was observed among strains at 2, 4, 6, 8, and 24 h (*p* < 0.0001), whereas no significant differences were detected at 0 and 48 h (*p* > 0.05). All strains were capable of reducing milk pH after 4 h, although none reached the threshold value of pH 5. A marked drop in pH was observed starting at 8 h, with a pronounced acidification between 24 and 48 h. Final pH values ranged from 4.32 ± 0.01 (CMLAB9) to 4.92 ± 0.01 (CMLAB12), confirming a strong acidification trend. In parallel, titratable acidity measurements revealed lactic acid concentrations between 0.71 and 1.02 g/L after 48 h, in line with efficient lactose fermentation. This acidification is critical for microbial safety, as a low pH environment (<4.5) is known to inhibit the growth of foodborne pathogens, including *Listeria monocytogenes* and *Staphylococcus aureus*, thereby supporting the potential use of these strains as bioprotective cultures in dairy matrices. Furthermore, enumeration of the total microbial load in inoculated milk confirmed the inhibitory effect of acidification. After 48 h of incubation, the total viable count remained stable or decreased in milk inoculated with the LAB strains, whereas an increase was observed in the uninoculated control samples. These results support the hypothesis that acidification, combined with the metabolic activity of selected *Lactococcus* strains, plays a dual role in enhancing food safety: lowering pH and exerting antagonistic effects on spoilage and pathogenic microorganisms.

### 3.9 LAB biopreservative activity and staphylococcal inhibition in raw meat

#### 3.9.1 Microbial load evolution in meat samples

**Figure 7:**
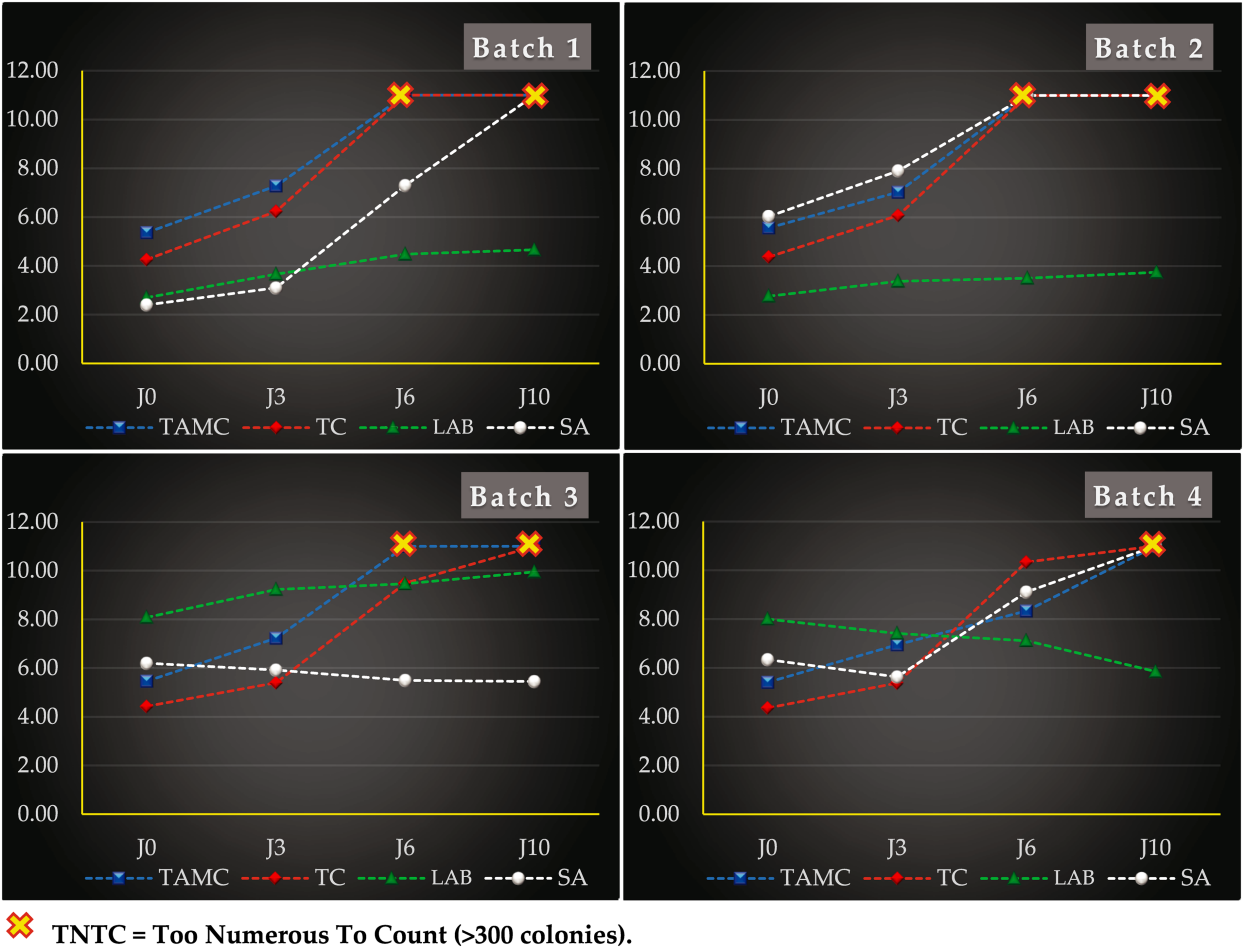
Microbial load evolution (expressed in log₁₀ CFU/g) of microbial groups in the different batches of fresh beef filets during 10 days of storage at 8 °C: (TAMC) Total aerobic mesophilic flora, (TC) Total coliforms, (LAB) Lactic Acid Bacteria, (SA) *Staphylococcus aureus*.

Figure 7 illustrates the evolution of the counts of total aerobic mesophilic flora, total coliforms, LAB, and *Staphylococcus aureus* on four experimental batches during storage under refrigeration conditions (0, 3, 6, and 10 days at 8 °C). A highly significant difference was observed among batches (*p* < 0.0001). In batch 1 (control), TAMC rose from 5.36 to 7.28 log₁₀ CFU/g within the first 3 days, while TC increased from 4.26 to 6.23 log₁₀ CFU/g. Both indicators reached peak levels by day 6.

Batch 2, inoculated with *Staphylococcus aureus* ATCC 25923 only, showed microbial trends comparable to batch 1, confirming the minimal influence of the pathogen on overall microbial proliferation. In contrast, batch 3, treated with LAB consortium MCP1, showed an increase in TAMC from 5.46 to 7.23 log₁₀ CFU/g by day 3, with a similar trend to batches 1 and 2. Notably, batch 4, treated with MCP2, reached a TAMC peak of 8.34 log₁₀ CFU/g at day 6.

Coliforms followed similar trends. On day 3, TC reached 5.40 log₁₀ CFU/g (batch 3) and 5.38 log₁₀ CFU/g (batch 4), peaking at 9.49 and 10.34 log₁₀ CFU/g, respectively, after 6 days.

#### 3.9.2 LAB growth kinetics in meat

Indigenous LAB in control samples (batch 1) increased from 2.70 to 4.66 log₁₀ CFU/g over 10 days. Interestingly, in batch 2, inoculated solely with *S. aureus*, LAB counts were reduced by approximately 0.91 log₁₀ CFU/g compared to the control, suggesting an antagonistic effect. In inoculated batches 3 and 4, LAB counts started near the expected inoculum load. After 10 days, batch 3 (MCP1) reached 9.95 log₁₀ CFU/g, while batch 4 (MCP2) showed a marked decrease, from 8.0 to 5.85 log₁₀ CFU/g, reflecting a progressive decline over time. Reductions of 1.82, 2.35, and 4.10 log₁₀ CFU/g were recorded on days 3, 6, and 10, respectively, compared to batch 3.

#### 3.9.3 Anti-staphylococcal activity of mixed LAB cultures

**Figure 8:**
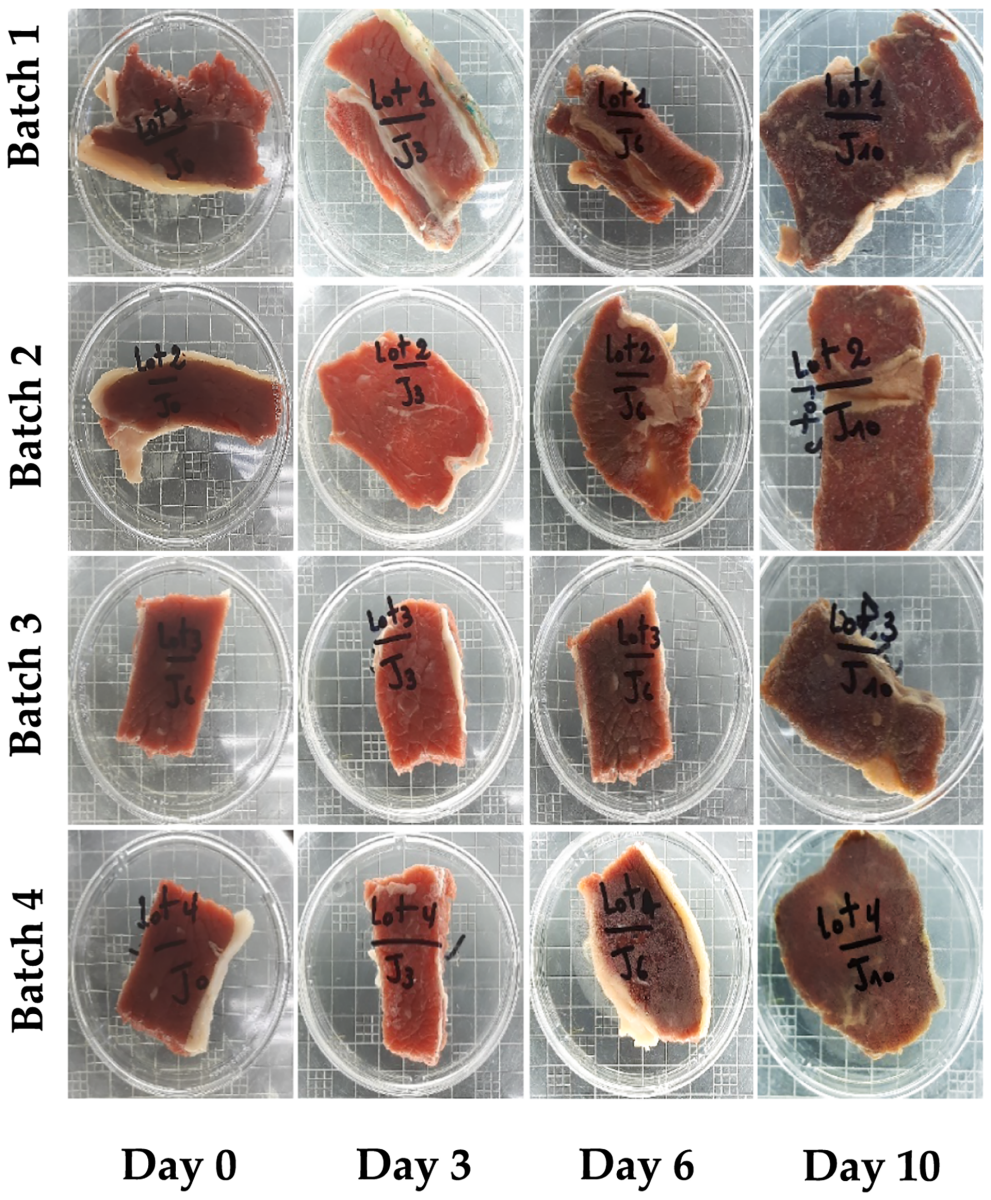
Changes in the qualitative visual appearance of fresh beef filets for the four experimental batches during the chilled storage period at 8 °C: (1) Control, (2) Inoculated with *Staphylococcus aureus*, (3) Inoculated with *S. aureus* and treated with mixed LAB culture MCP1, (4) Inoculated with *S. aureus* and treated with mixed LAB culture MCP2. Each time point (Day 0, 3, 6, and 10) represents an independent portion derived from the same initial sample to prevent cross-contamination and ensure microbiological integrity during storage and analysis.

In batch 1, natural contamination with *S. aureus* increased from 2.40 log₁₀ CFU/g to peak levels by day 10. In batch 2, deliberate inoculation with *S. aureus* (6.04 log₁₀ CFU/g) led to a rise to 7.91 log₁₀ CFU/g by day 3, peaking at day 6. In batch 3 (MCP1 + *S. aureus*), the pathogen count decreased from 6.20 to 5.45 log₁₀ CFU/g over 10 days, demonstrating a clear inhibitory effect. Conversely, batch 4 (MCP2 + *S. aureus*) showed only moderate inhibition: from 6.34 log₁₀ CFU/g on day 0 to 9.11 log₁₀ CFU/g on day 6, with a final count increase at day 10. A temporary inhibition phase was observed at day 3 (0.71 log₁₀ CFU/g reduction), followed by regrowth. In order to visually monitor the effect of different treatments on meat spoilage over time, photographic documentation was carried out throughout the storage period. As shown in Figure 8, the macroscopic changes in color and surface appearance among the different batches were evident as storage progressed. It is important to note that, to ensure microbiological integrity and avoid cross-contamination, each meat sample was portioned at the beginning of the experiment into independent aliquots, each designated for analysis and imaging at a specific time point. This means that the images captured at Days 0, 3, 6, and 10 represent different portions from the same initial sample, and not the same physical piece of meat over time. This methodological approach, while essential for analytical reliability, may give the visual impression of inconsistency in the photographed cuts.

## 4 Discussion

### 4.1 Safety and functional characterization of LAB strains

Meat and meat products are highly perishable due to their high water activity (aw), favorable pH, and rich nutritional content, which together pose a considerable challenge for preservation. In response, biopreservation, defined as the use of microorganisms or their metabolites, has gained momentum as a viable strategy. Lactic acid bacteria (LAB), in particular, have garnered substantial interest owing to their safety profile and longstanding use in traditional fermentations (37).

In this study, two mixed LAB cultures, composed of *Lactococcus lactis* subsp. *cremoris* strains, were evaluated for their biocontrol capacity against *Staphylococcus aureus* in fresh beef. The strains were first identified using MALDI-TOF MS, a rapid and reliable technique for LAB characterization (34). Three species were detected: *Lactococcus lactis*, *Lactobacillus lactis*, and *Enterococcus italicus*, with *L. lactis* being dominant. This species composition is in agreement with previous findings on LAB communities in camel milk (38–40).

Safety evaluations of LAB included antibiotic susceptibility testing and the assessment of gelatinase, DNase, and hemolytic activities, essential parameters to ensure non-toxicity (41, 42). Strains with any of these virulence-associated traits were excluded. In particular, the absence of hemolysis suggests non-virulent behavior, as hemolytic strains are often associated with pathogenicity (43). The ability to avoid the production of biogenic amines, known to exert toxic effects, was also considered a critical safety trait (23). Furthermore, gelatinase activity—a factor aiding pathogen spread via tissue degradation, was not observed in the safe strains (44).

Antibiotic susceptibility varied among the strains, highlighting the importance of individual evaluation. However, resistance in probiotics is not inherently hazardous unless it is transferable to pathogens or renders the strain untreatable in case of infection (45). Several studies have already validated the safety of *Lactococcus* and *Lactobacillus* strains (46–48). Notably, *Lactococcus lactis* has recently been confirmed as safe across multiple parameters, including antibiotic susceptibility and hemolytic activity (46). LAB are generally recognized as safe (GRAS) by the FDA and hold Qualified Presumption of Safety (QPS) status from EFSA (49).

The antimicrobial activity of these strains was assessed via inhibition assays against *S. aureus* ATCC 25923. Most strains demonstrated strong antibacterial activity, with CMLAB1 and CMLAB9 being the most potent. These properties are largely strain-specific. While lactic acid is the principal inhibitory metabolite, LAB also produce a diverse array of bioactive compounds—including acetic and formic acids, ethanol, hydrogen peroxide, bacteriocins, diacetyl, and less-characterized inhibitory molecules (50–52).

Additionally, the antioxidant potential of LAB was confirmed via ABTS radical scavenging assays. Strain CMLAB8 showed the highest antioxidant activity (81.3 ± 1.5%), consistent with prior studies (19, 30). LAB produce antioxidant substances such as exopolysaccharides, enzymes, peptides, and manganese ions, and they can bioconvert dietary precursors into bioactive compounds (53). These properties support their value in extending food shelf life and improving quality.

The strains used for the *in situ* challenge (CMLAB1, CMLAB9, CMLAB10, and CMLAB12) were confirmed by 16S rRNA sequencing as *L. lactis* subsp. *cremoris*, and the phylogenetic tree corroborated their identification, distinguishing them from *L. piscium*. These results are consistent with MALDI-TOF MS data and underscored the dominance of *L. lactis* in camel milk microbiota (40, 54). Members of the *Lactococcus*, *Lactobacillus*, and *Carnobacterium* genera remain the most widely used protective cultures in meat and seafood (55, 56).

LAB were found to reduce pH below 4.6, enhancing their antimicrobial efficacy. Their applications in the food industry range from starter cultures for fermentation to lyophilized additives in fresh or ready-to-eat meats (57). Importantly, mixed cultures like MCP1 and MCP2 exhibit synergistic inhibitory effects greater than those of individual strains (58, 59).

### 4.2 Biopreservative performance of mixed LAB cultures in meat samples

The mixed LAB cultures MCP1 and MCP2 were tested for their antimicrobial activity in raw beef samples over a 10-day storage period at 8 °C. The goal was to monitor their effects on *S. aureus*, total aerobic mesophilic counts (TAMC), total coliforms (TC), and LAB dynamics.

Compared to controls, MCP1 showed moderate inhibition of TAMC, while MCP2 exerted a more significant effect (*p* < 0.0001), achieving a 2.66 log₁₀ CFU/g reduction by day 6 (24.18% inhibition). For TC, both cultures were effective: MCP1 and MCP2 reduced counts by 1.51 and 0.66 log₁₀ CFU/g respectively, corresponding to 13.73 and 6% inhibition.

These results are consistent with other reports. For instance, *Lactobacillus sakei* and *L. curvatus* reduced microbial diversity and extended shelf life in vacuum-packed beef (60), while bacteriocin-producing *L. lactis* reduced TAMC by 4.87 log₁₀ CFU/g in stored meat (61). Similarly, *L. plantarum* improved microbiological quality in poultry meat, albeit with a rise in Enterobacteriaceae (62). In this study, both mixed cultures notably decreased TC (*p* < 0.0001), suggesting efficacy against Enterobacteriaceae.

LAB loads increased naturally in control samples, suggesting good adaptation to the meat matrix. However, in samples co-inoculated with *S. aureus*, LAB declined, indicating antagonism. While MCP1 maintained LAB growth during storage, MCP2 showed a significant decline, suggesting inferior adaptation. This divergence may explain the weaker inhibitory effect of MCP2. Literature confirms that LAB survival and efficacy can vary depending on food matrices and incorporation methods (63, 64).

MCP1 exhibited strong inhibition of *S. aureus*, with reductions of 0.75 log₁₀ CFU/g on day 1 and 5.55 log₁₀ CFU/g by day 10, corresponding to a 50.45% inhibition. In contrast, MCP2 showed only moderate inhibition (1.89 log₁₀ CFU/g or 17.18%) by day 6 and failed to suppress initial counts. This weaker response may be linked to MCP2’s limited growth in meat. Nevertheless, the differential effect was significant (*p* < 0.0001), highlighting MCP1’s superior biocontrol potential.

The observed microbial reductions are considered biologically significant. A decrease of this magnitude is generally correlated with a notable prolongation of the shelf life of chilled meats, as it delays the establishment of spoilage flora and limits the risk of pathogenic germ proliferation. The level of logarithmic reductions obtained is comparable to values previously reported in the literature.

*Ligilactobacillus salivarius* reduced *L. monocytogenes* and *Salmonella* spp. on chicken meat (65), and mixtures of *L. lactis*, *L. paracasei*, and *L. plantarum* reduced *L. monocytogenes* by 5 log₁₀ CFU/g in cooked ham (11). Similarly, *L. sakei* reduced *S. choleraesuis* in pork sausages (66), and *L. lactis* reduced *Brochothrix thermosphacta* and *Staphylococcus* spp. in pork (67).

Previous findings confirm that *L. lactis* can reduce *S. aureus* by 1 log CFU/g over 25 days (68) or 4.1 log CFU/mL in 24 h with bacteriocin-producing strains (69). These effects, even at low temperatures, underscore its usefulness for meat biopreservation (37, 70).

Recent data also highlight LAB’s ability to suppress *S. aureus* virulence by downregulating enterotoxin gene expression—suggesting an additional layer of safety in reducing foodborne illness (56, 71, 72).

However, methodological differences, such as food matrix, pathogen strain, LAB concentration, and storage conditions, limit direct comparisons across studies. Still, it is widely agreed that LAB show greater inhibition in food matrices than *in vitro* due to synergistic interactions with food components (73). Their action mechanisms include membrane disruption, inhibition of cell wall synthesis, interference with proton gradients, and ROS production (74, 75).

Furthermore, LAB are capable of synthesizing a wide range of antimicrobial molecules. These compounds include organic acids and hydrogen peroxide, as well as specialized metabolites such as bacteriocins, biosurfactants, and lipopeptides, thereby effectively contributing to the inhibition of the growth of pathogenic or spoilage microflora (76, 77).

### 4.3 Limitations and future perspectives

Although this study provided significant insights regarding the potential of LAB for the control of pathogens and the biopreservation of beef, it nonetheless has certain limitations. These notably concern the evaluation of the impact of LAB on the sensory properties of the matrix, as well as the study of their combination with different packaging systems. Consequently, future research perspectives should focus on confirming these results on a larger scale, establishing a metabolomic profiling of the compounds responsible for the observed inhibitory activity, and evaluating their efficacy in other food matrices. It would be particularly relevant to investigate the association of LAB with controlled-release systems or other mild preservation technologies (high pressure, modified atmosphere).

## 5 Conclusion

Staphylococcal food poisoning, caused by the thermostable enterotoxins of *Staphylococcus aureus*, remains a major global public health concern. This pathogen is characterized by high virulence, great adaptability, and increasing antibiotic resistance. In this context, lactic acid bacteria (LAB) constitute a natural and sustainable alternative to classical preservation methods, meeting the current demands of industry and consumers. In this study, two mixed cultures of *Lactococcus lactis* subsp. *cremoris*, isolated from raw camel milk and selected from an initial panel of 19 LAB strains. The selected cultures were tested in a refrigerated beef model to evaluate their ability to control *S. aureus* and other spoilage flora. The results confirmed the inhibitory potential of the two mixed cultures of LAB, both against *S. aureus* and in reducing the total aerobic mesophilic count (TAMC) and total coliforms (TC). Their good adaptation to the meat matrix and low temperatures highlights their value for biopreservation. This efficacy probably results from a synergy of mechanisms, including acidification, nutritional competition, and the production of antimicrobial metabolites. In conclusion, these two LAB cultures constitute promising bioprotective agents, offering a sustainable and acceptable approach to enhance the microbiological safety and quality of meat products.

## Data Availability

The original contributions presented in the study are included in the article/Supplementary material, further inquiries can be directed to the corresponding authors.
