# Supplementary material for: Camel milk Lactococcus lactis subsp. cremoris: a biocontrol agent against Staphylococcus aureus for fresh beef biopreservation
Source: Front Nutr. 2026 Jan 8;12:1683200. doi: 10.3389/fnut.2025.1683200 (PMC12829333; doi:10.3389/fnut.2025.1683200)
Supplement: Supplementary file 1 [file Table_1.docx]

Supplementary Table 1. Antibiotic susceptibility profiles of LAB strains assessed by the disk diffusion method.

|  |  |  | **Strains** | | | | | | | | | | | | | | | | | | |  |  |  |  |
| --- | --- | --- | --- | --- | --- | --- | --- | --- | --- | --- | --- | --- | --- | --- | --- | --- | --- | --- | --- | --- | --- | --- | --- | --- | --- |
| **Antibiotic tested** | **Concentration  (µg/disc)** | **Code** | **CMLAB1** | **CMLAB2** | **CMLAB3** | **CMLAB4** | **CMLAB5** | **CMLAB6** | **CMLAB7** | **CMLAB8** | **CMLAB9** | **CMLAB10** | **CMLAB11** | **CMLAB12** | **CMLAB13** | **CMLAB14** | **CMLAB15** | **CMLAB16** | **CMLAB17** | **CMLAB18** | **CMLAB19** |  | **S (%)** | **I (%)** | **R (%)** |
| Lincomycin | 15µg | L15 | I | S | S | I | I | S | I | R | S | S | I | S | I | S | I | I | R | I | R |  | 36,8 | 47,4 | 15,8 |
| Nalidixic acid | 30µg | NA30 | S | S | R | R | R | R | S | S | R | I | R | R | R | R | R | R | S | S | S |  | 36,8 | 5,3 | 57,9 |
| Penicillin | 10 µg | P10 | S | I | S | R | S | S | R | R | I | S | S | I | I | S | S | S | R | R | I |  | 47,4 | 26,3 | 26,3 |
| Streptomycin | 300µg | S300 | I | I | S | R | I | R | S | R | R | R | R | I | R | I | R | I | S | S | S |  | 26,3 | 31,6 | 42,1 |
| Gentamicin | 10µg | CN10 | I | R | R | R | R | R | R | R | R | R | R | R | R | R | R | R | I | S | I |  | 5,3 | 15,8 | 78,9 |
| Ampicillin | 10µg | AM10 | S | S | S | R | S | S | R | R | I | S | S | I | I | I | S | I | R | R | R |  | 42,1 | 26,3 | 31,6 |
| Cephalothin | 30µg | KF30 | S | R | I | S | S | I | R | R | R | R | I | R | I | I | I | I | R | R | R |  | 15,8 | 36,8 | 47,4 |
| Erythromycin | 15µg | E15 | I | I | I | S | S | I | R | R | I | S | R | I | R | I | R | R | R | R | R |  | 15,8 | 36,8 | 47,4 |
| Ciprofloxacin | 5µg | CIP5 | R | I | I | R | S | R | S | R | R | R | R | I | R | R | R | R | S | S | S |  | 26,3 | 15,8 | 57,9 |
| Amoxicillin | 25µg | AX25 | R | S | S | S | S | S | R | R | I | S | I | S | R | I | I | R | R | R | R |  | 36,8 | 21,1 | 42,1 |
| Tetracycline | 30µg | TE30 | S | I | S | S | R | S | R | R | S | S | I | S | R | I | R | I | R | I | R |  | 36,8 | 26,3 | 36,8 |
| Kanamycin | 30µg | K30 | R | S | R | I | R | R | R | S | I | I | R | R | R | R | R | S | I | S | I |  | 21,1 | 26,3 | 52,6 |
| Azithromycin | 15µg | AZM15 | I | I | S | S | I | I | I | R | I | I | I | S | R | I | I | R | R | R | I |  | 15,8 | 57,9 | 26,3 |

Antibiotic susceptibility was evaluated according to the diameter of the inhibition zone (ZI) around the disc: S = Sensitive (ZI ≥ 21 mm), I = Intermediate (16 mm ≤ ZI ≤ 20 mm), R = Resistant (ZI ≤ 15 mm). Each strain was tested against 13 antibiotics, and results are reported as categorical interpretations (S, I, R).
